# Twelve Months of Abdominal and Vaginal Section*Presidential address delivered at the annual meeting of the Gynæcological Society of Chicago, October 19, 1888.

**Published:** 1888-11

**Authors:** Henry T. Byford


The Medical Journal and Examiner.

VOLUME LVII. NOVEMBER, 1888. NUMBER 5.

### TWELVE MONTHS OF ABDOMINAL AND VAGINAL SECTION.*

BY HENRY T. BYFORD, M. D.

GENTLEMEN: I have chosen to lay before you on this occasion an abstract of my work in abdominal and vaginal section during the year in which I have served you as president. As a detailed report of forty-eight cases would take up too much of your time, I have furnished each of you with a statistical table of them for your inspection, and my remarks will be chiefly of a critical and explanatory nature.

First, as to the percentage of deaths: A death-rate of 17 per centum can not be considered an unusually large one for operations in which the abdominal cavity is opened for all sorts of pelvic growths as they are met with in gynæcologic practice, including malignant and almost hopeless cases. Yet this mortality is above twice what it ought to have been in this series, or ought to be in almost any such series.

The two deaths from hæmorrhage should not have occurred. I suppose that almost every laparatomist of any experience has lost one or two patients from hæmorrhage, and has needed one or both of these accidents to teach him how to tie the pedicle. And until this shall be adequately taught somewhere, I suppose that almost every coming laparotomist will lose a patient or two in this manner.

The teaching that the pedicle should always be transfixed has diminished the frequency of hæmorrhage, but has not prevented its occurrence. The kind of knot cannot be made to solve the question, for hæmorrhage has occurred with all kinds of knots. In both of my cases of death from hæmorrhage I followed the directions usually given in the books and employed in practice, and I attribute the death of one, if not both, to my faithfulness in following out such advice. I transfixed the pedicle in a non-vascular place, and, although I tied as tightly as I could, my patient was almost pulseless from internal hæmorrhage four hours afterward.

I will substantiate the position taken by a few quotations. First, from Heger and Kaltenbach (Operative Gynakologie, third ed., 1880, p. 273): "The pedicle, held between two fingers, should be transfixed at a *non-vascular** place, . . . . a double thread drawn through and each *half* of the pedicle tied by itself, without crossing the threads. . . . The threads, at the tying of the first knots are always put through twice (surgical knot), and drawn with a gradually increasing force until they have made a deep and permanent furrow; thereupon, a second, and even a third, knot is tied. Below the partial ligatures we always put a ligature about the whole pedicle."

* Presidential address delivered at the annual meeting of the Gynæcological Society of Chicago, October 19, 1888.

* Italics in these quotations are mine.

Next from Olshausen (Handbuch der Frauenkrankheiten, Billroth & Lueke, 1886, vol. ii., p. 544): "It is generally accepted that thick pedicles must be tied in two, three, or, exceptionally, four portions. With the ordinary and quite efficient division in two *halves*, one separates the tissues in the *middle*, thinnest portion at a *non-vascular* place. . . . In the *half* on one side lies the tube; in the other, the ovarian ligament." The following is Greig Smith's description (Abdominal Surgery, 1887, p. 167): "The ligature is placed double by transfixing with a blunt needle. The inner pedicle contains the utero-ovarian ligament, the Fallopian tube somewhere near its isthmus, the spermatic artery and its veins, and the small branch which accompanies the Fallopian tube. The outer ligature lies at the retiring angle where the infundibulo-pelvic and infradibulo-ovarian ligaments meet, takes its *half* of the mesovarium, and also constricts the spermatic artery."

It will be noticed in these quotations that the pedicle is usually to be tied in *halves* and transfixed in a *non-vascular* point, which is generally a weak one. In both of my fatal cases I transfixed through a non-vascular portion, and in the one case (ovariotomy), I drew upon the silk as tightly as I could, while in the other (oöphorectomy), I used Tait's knot, and thought I got it as tight as possible. I opened the abdomen before death in both cases and found that the pedicle of the ovarian tumor had partly slipped out of the ligature, and that the other had not been sufficiently secured.

I now use the following method, with a view to avoiding such accidents, and have not found it wanting in safety: I hold the pedicle between the thumb and forefinger of the left hand so that the Fallopian tube, well drawn out, lies on the same side with (and against) the ovarian ligament. The mesovarium lies against the mesosalpinx. I pass the double-threaded needle with one thrust through the inner or mesenteric edges of both the Fallopian tube and ovarian ligament, thus getting the ligatures fixed at two firm points. I then hand the pedicle to an assistant and tie the tube and ovarian ligament with one of the threads, and the remainder of the pedicle, which is much more than *half*, with the other; and then the whole pedicle *en masse* with one of the same threads, preferably the one first tied. While drawing the ligatures tight with the first or surgical knot of each ligature, I keep on pulling at the knot while the traction upon the pedicle is relaxed by the assistant. This is necessary to secure complete collapse and permanent constriction of the tissues. When the pedicle is unusually short and inelastic I transfix, as just stated, but, in addition, carry both threads before tying through a fold of the pedicle at the side opposite the Fallopian tube and ovarian ligament, and thus have a hold at three peripheral points of the pedicle, and am insured against slipping of the ligature. To get a firm hold for the ligature, which is inconsistent with tying the pedicle in *halves*, and to relax the pedicle while tying, so that it may be made to collapse completely, are, it seems to me, points that can not be neglected with safety in any case, and which ought to be more fully explained in the books. Fleshy pedicles must, of course, be ligated in small portions, and are not referred to in what I said above.

The death from heart-failure (exploratory excision) ought not to have occurred, for either I ought not to have operated, or, having done so, should have pushed stimulants, nourishment, massage, etc., more vigorously. At present, whenever, after the first two or three days' fasting, the pulse becomes soft and compressible, I give some stimulants, particularly between the hours of 2 and 8 a. m., at which time the temperature is lowest and the circulation feeblest. Rectal alimentation is, of course, employed, but is always used with care for fear of inducing rectal irritation.

The two deaths from abdominal hysterectomy occurred in patients who should not have been operated upon, because the condition proved to be such that recovery after an operation was impossible. Yet, as they were both doomed to a speedy, painful death if left without interference, and were anxious for an operation, I could not see my way clear to refusing them what seemed a last chance. It is also certain that the other fatal ovariotomy ought not to have been performed, since cancerous deposits existed elsewhere than in the ovary. Let us hope that in the future, either cases like these three will be operated upon sooner, or an increased experience will enable us to diagnose more completely and determine with greater accuracy just when it becomes our duty to abandon the patient to her disease.

Had I known how to tie a pedicle, and had I known better how to select my cases, six of these seven deaths would not have occurred, and the mortality for the series would have been less than 3 per centum. But it is, of course, unjustifiable and cowardly for a surgeon to reject all unpromising cases, when he knows that they must speedily die if not relieved by an operation.

It will be seen that all of the deaths took place among the twenty-seven abdominal sections, and none among the twenty-one vaginal sections.

In preparing the patients for the operations I usually give five or six grains of blue-mass on the second night before, and follow it in the morning by a saline aperient. The mercurial leaves the secretions in a more healthy state than other laxatives, and better promotes absorbtion of the gases. Unless that works too thoroughly, an enema is given the evening before the operation, of two ounces of glycerine with four of water, and a plain water-enema on the morning of the operation. From one to two ounces of brandy or whisky are given half an hour before the anæsthetic is administered. I prefer cloroform, but use ether because of the difficulty in getting an assistant who is accustomed to use the former.

In the after-treatment no morphine is given, except for diarrhœa, or occasionally one dose immediately after the operation, for excessive nausea or restlessness. Pain, due to intestinal peristalsis, is treated by aromatics, the rectal tube, and the glycerine-and-water enema.

Vomiting after the operation, even though it continue for two or three days, is not considered as of serious consequence. But secondary vomiting, commencing gently after the first has subsided, with regurgitation at pretty regular intervals and gradually increasing in severity, is regarded as the forerunner of intestestinal obstruction or paralysis, or possibly of peritonitis. I then make haste to administer a saline aperient before the nausea becomes so severe as to prevent its retention. One drachm of the granular effervescing citrate of magnesia is given every hour until flatus or feces pass the anus, or until its action can be felt in the bowels by the patient. After this has occurred, or if it does not occur after ten or twelve drachms have been retained, the usual glycerine enema is given, and repeated, if neccessary, with the addition of a drachm of spirits of turpentine. In case the saline aperient produces much pain it is discontinued and the enemas relied upon exclusively. When intestinal gases commence to pass off without the aid of the rectal tube, I consider the danger of intestinal obstruction or paralysis over for the time, and wait for other symptoms, or until near the end of the first week, before further disturbing the bowels. The effect of the magnesia should be to increase the comfort of the patient and diminish her pain; if it does not do so, it is not, as a rule, indicated.

In operating, I usually try to obtain as thorough asepsis as practicable, but, as a rule, do not consider its perfect attainment possible. I endeavor to operate so that my patient will recover, even if some of the ordinary septic germs of the atmosphere be introduced into the abdominal cavity, and seldom feel certain of completely excluding them, except in simplest cases with small incisions. The part of the peritoneum with which I consider it most dangerous to deal is that which covers the intestines. I touch them only when absolutely necessary, and keep them as nearly constantly out of view or exposure to the air, by a covering of omentum, sponge, or aseptic cloth, as possible. I think the chances for recovery in any given properly performed abdominal or vaginal section will be bad almost in proportion as the intestinal coverings are injured or exposed to the air.

Finally, gentlemen, I will close with remarking that the way of the laparotomist, like that of the transgressor, is hard. The lot of the ideal antiseptist is unusually so, especially if he have that fear of sepsis that goes with the so-called aseptic conscience. At a recent abdominal section I had to make a large incision and spend some time in an attempt to control hæmorrhage in the pelvis. About the time I had got the abdominal cavity open an invited guest came in, stating that, through a mistake of the messenger, he had just received my invitation and had started for the hospital immediately. He was, of course, unprepared. When I called for ligatures he, considering himself an assistant, made a grab for them. I had him put them down, and gave him a short lecture. Yet in a few minutes he had his hand upon the edge of the incision, holding an instrument. After the operation I found among my instruments a pair of hæmostatic forceps, which he had taken out of his pocket-case after I had used my last one. Upon leaving he informed me that the case had been an instructive one to him, and, although the patient got well without a bad symptom, it was also an instructive one to me.

### Abdominal Sections for the Removal of Ovarian Tumors.

| No. | Name, and Date. | Age. | Married or Single. | No. of Children. | No. of Tappings. | Size and Nature of Tumor. | One or both Ovaries. | Adhesions. | Treatment of Pedicle. | Drainage. | Complications. | Hospital or Private. | Result. | Reported Elsewhere. | Remarks. |
|---|---|---|---|---|---|---|---|---|---|---|---|---|---|---|---|
| 1 | Mrs. P—th. July 14, 1887. | 40 | M | Multipara. | 1 for ascites | Carcinoma of right ovary size of man's head, cyst of left ovary size of fist. | Both | Extensive, to right abdominal wall | Tied with silk and dropped | Yes, long rubber tube | Ascites........... | St. Luke's Hospital | D. in 10 hrs | No | Died of shock and exhaustion. |
| 2 | Mrs. Tho—s. Aug. 30, 1887. | 27 | M | 4 | 0 | Dermoid cyst, size of large goose egg. | Both | No | Tied with silk and dropped | No | Fibro-myoma of fundus uteri, size of small orange. | Woman's Hospital | R | No | Uneventful recovery. |
| 3 | Mrs. W—sh. Nov. 19, 1887. | 48 | M | Multipara. | 0 | Monocyst, size of man's head, filled with chocolate-colored fluid. | One | Extensive omental | Tied with silk and dropped | No | Adherent, omentum indurated, size and shape of hand | St. Luke's Hospital | R | No | Removed thickened omentum after a multiple juniper catgut ligature. |
| 4 | Mrs. —. Jan. 11, 1888. | 40 | M | 0 | 0 | Monocyst, containing 3 pints. | One, right | No | Tied with silk and dropped | No | Urethritis, cystitis, insanity. | St. Luke's Hospital | R | No | Got up at beginning of 5th day, changed nightgown, put on skirt, and was found at the door twenty feet away. Next day temperature 98 2-5 to 99° Fahr. Got up again on 10th day. Recovery rapid and uninterrupted. |
| 5 | Mrs. P—s. Jan. 31, 1888. | 23 | M | Twins. | 0 | Polycyst, larger than a man's head | One, left | Slight | Tied with silk and dropped | No | Hæmorrhage from pedicle. | St. Luke's Hospital | D. in 27 hrs | No | Died of hæmorrhage. Opened abdomen about six hours after and found that the ligature had slipped. Abdominal cavity full of blood. Sewed up broad ligament. Transfusion of saline sol. and afterward of blood. |
| 6 | Mrs. S—l. May 29, 1888. | 28 | M | 2 | 0 | Dermoid cyst, size of woman's head. | Both | No | Tied with silk and dropped | Yes, for 3 days, glass tube | Metritis.......... | Woman's Hospital | R | No | Escape of fluid from tumor into incision. Washed out abdomen with warm water. Smooth recovery. |

### Vaginal Sections for the Removal of Ovarian Tumors.

| No. | Name, and Date. | Age. | Married or Single. | No. of Children. | Size and Nature of Tumor. | One or Both Ovaries. | Adhesions. | Treatment of Pedicle. | Drainage. | Complications. | Hospital or Private. | Result. | Reported Elsewhere. | Remarks. |
|---|---|---|---|---|---|---|---|---|---|---|---|---|---|---|
| 1 | Mrs. M—m. Aug. 29, 1887. | 40 | M | 8 | Monocyst size of smal l egg — long pedicle. | Both | None ...... | Tied and dropped. | Yes, 24 hours | Menorrhagia, retroversion, invalidism. | St. Luke's Hospital | R | Am. Journal Obst. April, 1888 | Tamponed uterus in position for 2 days. Cured retroversion and symptoms. |
| 2 | Miss R—e. Oct. 2, 1887. | 24 | S | 0 | Dermoid cyst size of walnut, right. | Both | Uterus and appendages matted together. | Tied and dropped. | Yes, 40 hours | Left pyo-salpinx. Patient bedridden | Woman's Hospital | R | Am. Journal of Obst., April, 1888, 9, v. | Abscess developed in the 4th week and discharged into vagina and rectum. Is now improving in health, is menstruating. |

### Abdominal Section for Removal of Uterine Appendages not the Seat of Tumor

| No. | Name, and Date of Operation. | Age | Married or Single. | No. of Children. | Pathological Condition or Symptoms necessitating Operation. | Duration of Disease. | What Removed. | Adhesions. | Treatment of Pedicle. | Drainage. | Hospital or Private. | Recovery or Death. | Complications before or after Operation. | Elsewhere Reported. | Remarks—Effect of Operation upon condition requiring it. |
|---|---|---|---|---|---|---|---|---|---|---|---|---|---|---|---|
| 1 | Miss McL. July 21, 1887. | 24 | S | ........ | Hystero-epilepsy. Follicular enlargement of both ovaries (slight). | .......... | Both ovaries, with tubes | No | Tied and dropped | No | Woman's Hospital | R | None | No | Troubled since operation with dark, offensive discharge from the uterus. Symptoms were relieved for a time, but since as bad as ever at times, and better at times. |
| 2 | Mrs. A. R. R—ll Aug. 10, 1887. | 23 | M | 1 child, 1 misc. at 6 mo. | Small fibroid of fundus uteri. Epilepsy, numerous severe fits daily, near menstrual period. | Several years | Both ovaries, with tubes | No | Tied and dropped | No | Woman's Hospital | R | Pelvic hematocele size of large orange, 3 weeks after operation produced by eating bananas; absorbed in 3½ months. | No | Epilepsy cured for a few weeks, then returned; but not as bad as before operation, and is now improving. |
| 3 | Mrs. McK—a. Aug. 11, 1887. | 26 | W | Three | Periodic hysterical mania, worse at menstrual periods. Enlarged ovaries. | Seven years | Both ovaries, with tubes | No | Tied and dropped | No | Woman's Hospital | R | Retroplacement and anteflexion of uterus. | No | Complete cure to date. Had two slight attacks soon after operation —none since. In good health. |
| 4 | Mrs. Kr—r. Aug. 29, 1887. | 19 | S | ........ | Chronic interstitial ovaritis and enlargement. Pain. (See remarks). | Six years, since first menstrual period | Both ovaries, with tubes | No | Tied and dropped | No | St. Luke's Hospital | R | Premature menopause. Patient plethoric | Chicago Medical Society, 1887 | Complete cure. Ovaries nearly four times natural size and each converted into a multitude of cysts size of peas, none of which projected from the surface. |
| 5 | Mrs. S—nd. Sept. 8, 1887. | 19 | W | One, a year before | L. hæmato-salpinx. Both ovaries about 4 times normal size and inflamed. | One year | Both ovaries, with tubes | Extensive, on both sides. | Tied and dropped | No | St. Luke's Hospital | R | Slight attack of phlebitis (r) following operation. Defecation painful for 2 months after. Cervical stenosis from anteflexion, with uterine colic. | No | Condition originated in puerperal septicæmia. Now in fair health and improving, but has a small, tender lump on right broad ligament. |
| 6 | Miss Fr—ll. Sept. 26, 1887. | 19 | S | ........ | Ovaritis of long standing. Unimproved by p'longed treatment. | Uncertain | Right ovary and tube | No | Tait's knot | No | St. Luke's Hospital | D. in 36 hours | Hæmorrhage from pedicle. | No | Abdominal cavity opened 19 hours after operation, after transfusion of nearly Oil of saline fluid, and found ligature partly off of stump, abdominal cavity full of blood-clots. |
| 7 | Mrs. L—n. Oct. 6, 1887. | 51 | M | Multip. | Cyst of right broad ligament. Invalidism unimproved by treatment. Small cyst projecting f'm surface of left ovary | Several years | Left ovary and tube | Cyst on left ovary firmly adherent, causing free bleeding. | Tied and dropped | Glass tube for 24 h'rs, iodoform gauze 48 hours longer | St. Luke's Hospital | R | None | No | Patient slightly improved. Drew the fluid from cyst of broad ligament by aspiration per vaginam Nov. 7, 1887, at the place of its projection over the cul de sac. Abdominal walls were so very fat that a large abdominal incision would have been necessary even to aspirate the cyst from above. Intestines full and tense. |

| No. | Name, and Date of Operation. | Age. | Married or Single. | No. of Children. | Pathological Condition or Symptoms necessitating Operation. | Duration of Disease. | What removed. | Adhesions. | Treatment of Pedicle. | Drainage. | Hospital or Private. | Recovery or Death. | Complications before or after Operation. | Elsewhere reported. | Remarks—Effect of Operation upon condition requiring it. |
|---|---|---|---|---|---|---|---|---|---|---|---|---|---|---|---|
| 8 | Mrs. S—dy. Oct. 14, 1887. | 37 | Wid. | 0 | Left hydrosalpinx. Enlarged cystic ovaries, chronic invalidism, unimproved by treatment. | 14 years | Both ovaries with tubes. | No | Tied and dropped. | No | Woman's Hospital. | R | Small hernia followed but gives no trouble. Endometritis before operation. | Chicago Gyn. Soc., Nov. 18, 1887. | Slow improvement after operation. |
| 9 | Mrs. K—n. Dec. 19, 1887. | 22 | M | 0 | Enlarged cystic ovaries. Interstitial salpingitis. Distension of veins of right broad ligament. Excruciating dysmenorrhœa. | 2½ years | Both ovaries with tubes. | Universal and firm. | Tied and dropped. | Yes, glass tube. | Woman's Hospital. | D. in 3½ days. | Persistent bloody oozing from drainage tube. Obstruction of bowels. | No | Death caused by obstruction of bowels with pelvic peritonitis. Highest temp. during first 3 days, 101 2-5° F.; before death, 102 2-5°. |
| 10 | Ellen A—n. Jan. 4, 1888. | 24 | S | ....... | Congenital anteflexion. Incurable dysmenorrhœa. Slightest dilatation followed by inflammatory reaction. | Since first menstrual period | Both ovaries with tubes. | No | Tied and dropped. | Yes, glass tube. | St. Luke's Hospital. | R | Losing mental power; acquiring opium habit. Becoming fast a physical wreck. | No | Recovery is complete. Now supports her aged mother. |
| 11 | Kate B—e. Jan. 16, 1888. | 24 | S | ........ | Epilepsy at or near menstrual periods. Loss of mental power, slight cystic enlargement of L. ovary. | 2½ years | Both ovaries with tubes. | No | Tied and dropped. | No | St. Luke's Hospital. | R | Epilepsy so far cured. Has an exudate size of end of thumb about right stump, preventing her from working. | No | Has menstruated regularly for past 3 months. Still complains of mental weakness, kept up perhaps by irritation about right stump. |
| 12 | Mrs. J—n. Jan. 22, 1888 | 23 | M | 1 Premature. | Double ovaritis and salpingitis, with recurrent acute attack. Right hematosalpinx. | Since birth of child | Both ovaries with tubes. | Extensive and firm. | Tied and dropped. | No | Woman's Hospital. | R | Followed in three months by rheumatic arthritis. Endometritis chronic before and after operation. | No | Gradual progressive recovery from all but endometritis. |
| 13 | Miss McF—n. March 26, 1888 | 35 | S | ........ | Soft fibro-myoma of fundus uteri size of goose egg with persistent incurable pelvic pains. Slight menorrhagia. | Symptoms for several years | Both ovaries with tubes. | No | Tied and dropped. | No | St. Luke's Hospital. | R | .................... | No | Left hospital complaining slightly of pelvic pains. Menses, which were rather profuse, had not returned. |
| 14 | Ida H—h. May 21, 1888. | 22 | S | ........ | Congenital anteflexion, with dysmenorrhœa and pelvic pains, increasing in spite of many months of treatment. | 5 years. Soon after menses appeared | Both ovaries with tubes. | No | Tied and dropped. | No | Woman's Hospital. | R | .................... | Chicago Medical Society, May 21, 1888. | Tenderness about one stump for about 3 months. Improving. Menses have not returned. |
| 15 | Miss P—w. June 26, 1888. | 25 | S | ........ | Dysmenorrhœa. Incurable pelvic pains Failure of mental power. Enlarged ovaries. | 7 years | Both ovaries with tubes. | No | Tied and dropped. | No | Woman's Hospital. | R | Cervical endometritis. Bluish softened ulcerated os. | No | Ovaries cystic and four times their natural size. Left hospital improved. |

### Vaginal Section for Removal of Uterine Appendages not the Seat of Tumor.

| No. | Name, and Date of Operation. | Age. | Married or Single. | No. of Children. | Pathological Condition or Symptoms necessitating Operation. | Duration of Disease. | What Removed. | Adhesions. | Treatment of Pedicle. | Drainage. | Hospital or Private. | Recovery or Death. | Complications before or after Operation. | Effect of Operation upon Condition requiring it. | Remarks. | Elsewhere Reported. |
|---|---|---|---|---|---|---|---|---|---|---|---|---|---|---|---|---|
| 1 | Mrs. E. D—ty. July 30, 1887. | 35 | M | Three | Hysterical mania and cephalalgia. Husband left her on account of supposed insanity. | Several years | Both ovaries and tubes | No | Tied and dropped | Yes, 24 hours | Woman's Hospital | R | Addicted to opium habit before operation. Retroversion. | Cured of mania, cephalalgia and opium habit. | Last attack of cephalalgia and mania was the day before the operation. | Am. Journal Obst. April, '88. |
| 2 | Mrs. N—n. July 31, 1887. | 31 | M | One, at 8 months | Oöphoritis, salpingitis. Right hydrosalpinx. Husband thought she was losing her mind. Several years' treatment without relief. Acute attacks. Unable to work. | Many years dating from confinement | R. ovary and tube. Loosened adhesions of left ovary. | Universal and firm | Tied and dropped | Yes. 24 hours | St. Luke's Hospital | R | Retroversion and endometritis before operation. Slight phlebitis on right side after operation. | Gradual progressive relief. Great gain in flesh. Able to do her housework. Mental symptoms cured. | Left ovary and tube should have been removed. | Am. Journal Obst. April, '88. |
| 3 | Mrs. S—r. Aug. 11, 1887. | 42 | M | Three | R. ovary enlarged and neuralgic, lying in recto-uterine pouch. Bedridden. Mental condition bordering on insanity. Failure of treatment. | Two years | Both ovaries and tubes | No | Tied and dropped | Yes, 24 hours | Woman's Hospital | R | Had been in asylum for an attack of insanity. Retroflexion. | Gradual uninterrupted recovery, almost complete when last heard from. | ................ | Am. Journal Obst. April, '88. |
| 4 | Mrs. Annie C. Aug. 17, 1887. | 24 | M | Two | Hæmatoma of R. ovary size of hickory nut. Bilateral ovaritis and salpingitis. Bedridden 3 years. | Three years | Both ovaries and tubes | Universal and firm. (See remarks.) | Tied and dropped | Yes, two days | St. Luke's Hospital | R | ................ | Gradual recovery. Examined in July, 1888, and found cure complete. | A porton of right tube left. | Am. Journal Obst. April, '88. |
| 5 | Mrs. C—n. Sept. 8, 1887. | 43 | M | One, 20 years old | R. ovary in recto-uterine pouch. L. ovary enlarged and cystic. L. tube hypertrophied. | 20 years | Both ovaries and tubes | No | Tied and dropped | Yes, 24 hours | St. Luke's Hospital | R | Light anteflexion. | Able to work out two months after operation. | ................ | Am. Journal Obst. April, '88. |
| 6 | Mrs. J—n. Sept. 26, 1887. | 34 | M | One | R. ovary in recto-uterine pouch. Both enlarged and cystic. | Since birth of child | Both ovaries and tubes | No | Tied and dropped | Yes, 48 hours | St. Luke's Hospital | R | Retroversion. | Rapid complete relief. | Retroversion cured by tamponing after operation. | Am. Journal Obst. April, '88. |
| 7 | Mrs. C. E. F. Oct. 18, 1887. | 28 | M | wo | R. ovary enlarged and in recto-uterine pouch. Inability to be on feet any length of time. | Four years | Right ovary and tube | No | Tied and dropped | Yes, 24 hours. | Woman's Hospital | R | Retroversion. | Rapid cure. | Retroversion cured. | Am. Journal Obst. April, '88. |
| 8 | Mrs. J—n. Jan. 14, 1888. | 42 | M | One, 16 years old | Chronic inflammation of appendages. Frequent acute attacks. R. ovary enlarged and cystic. | 16 years | Both ovaries and tubes | No | Tied and dropped | Yes, 30 hours. | Woman's Hospital | R | Retroversion before, pneumonia during, convalescence. | Relieved until the retroversion recurred two months after operation while going up stairs. Symptoms then recurred and have improved slowly since. | Uterus tamponed in position for 2½ days and remained in fair position for two months. | Am. Journal Obst. April, '88. |
| 9 | Mrs. H—d. Feb. 6, 1888. | 22 | M | One, 2½ yrs. old | R. ovary size of small hen's egg, prolapsed. Pain and disability. Failure of treatment. | 2½ years | Right ovary and tube | No | Tied and dropped | Yes, 24 hours. | St. Luke's Hospital | R | ................ | Immediate, complete and permanent relief. | ................ | Am. Journal Obst. April, '88. |

### Vaginal Section for Removal of Uterine Appendages not the Seat of Tumor—Concluded.

| No. | Name, and Date of Operation. | Age. | Married or Single. | No. of Children. | Pathological Condition or Symptoms necessitating Operation | Duration of Disease. | What Removed. | Adhesions. | Treatment of Pedicle. | Drainage. | Hospital or Private. | Recovery or Death. | Complications before or after Operation. | Effect of Operation upon Condition requiring it. | Elsewhere Reported. |
|---|---|---|---|---|---|---|---|---|---|---|---|---|---|---|---|
| 10 | Mrs. J. E. D. Feb. 16, 1888. | 26 | M | 0 | Left tube contained 4 ounces of serum; Left ovary half an ounce; Right ovary and tube enlarged and prolapsed | Several years. | Both ovaries and tubes | On left side. | Tied and dropped | Yes, 30 hrs. | Private | R | Retroversion before. Abscess in recto-uterine pouch, due to a small effusion of blood after operation. | Patient passed from observation. | Am. Journal Obst., Apr., 1888. |
| 11 | Mrs. J. H. E. Apr. 21, 1888. | 28 | M | 0 | L. hæmatosalpinx. Left ovary 4 times natural size. R. appendages diseased. Prolonged invalidism. | 12 years, since a fall. | Both ovaries and tubes | Extensive and firm on both sides. | Tied and dropped | Yes, 48 hrs. | Woman's Hospital | R | Fracture of coccyx. Retroversion. Small abscess in cellular tissue about stitches discharged ten days after the operation and gave no more trouble | Gradual improvement. Feels better than before operation. | No |
| 12 | Mrs. Mc E. Apr. 26, 1888. | 33 | M | 1 | Both ovaries enlarged, L. in recto-uterine pouch. Both tubes indurated and occluded. Persistent incurable pelvic pains. | Since miscarriage 5 yrs. ago. | Both ovaries and tubes | Slight of left tube. | Tied and dropped | Yes, 32 hrs. | Woman's Hospital | R | Retroversion. Exudate, size of chestnut, was found at site of L. stump 3 weeks after operation, which was rapidly absorbed. | Complete relief for three weeks, when the temp. went up to 100° F., and severe pains came on. Patient felt too well and exercised too much. Improving rapidly when she left hospital. | No |

### Abdominal Hysterectomy.

| No. | Name, and Date of Operation. | Age. | Married or Single. | No. of Children. | Pathological Condition and Symptoms necessitating Operation. | Duration of Disease. | Nature of Operation. | Adhesions. | Drainage. | Hospital or Private. | Recovery or Death. | Complications before and after. | Remarks and subsequent History. | Elsewhere Reported. |
|---|---|---|---|---|---|---|---|---|---|---|---|---|---|---|
| 1 | Mrs. W—n. Aug 4, 1787. | 40 | M | 4 and 4 misc. | Fibro-sarcoma of uterus size of man's head. Pain and rapid growth. | 6 years. | Amputation and fixation of stump in wound with use of elastic ligature. | Intestinal all over it; also attached to pelvic wall. | Yes. | Woman's Hospital. | Died from exhaustion in 36 hours | Unsuccessful attempt at removal had been made 2 months before. | Stump was size of a man's thigh. Had been diagnosed to be a fibro-myoma. | Chicago Gyn. Soc., Nov., 1888. |
| 2 | Mrs. E—tt Jan 2, 1888. | 34 | M | 1 | Fibro-cystic myoma size of uterus at 9 mo. Half pint of watery fluid in right tube. Left tube contained a quart and had grown down between folds of broad ligament to pelvic floor. | 7 years. | Amputation, treatment of pedicle after Schröder's (intraperitoneal). Left tube could not be removed, and was stitched into external wound. | L. tube adherent to tissues at base of broad lig. | Double. One tube in abdominal cavity, another in L. Fallopian tube. | Woman's Hospital. | Died of exhaustion in 48 hours with commencing rise of temp | Broad lig. drawn up over tumor so as to render ligation almost impossible. | .................. | No |
| 3 | Mary N—l. May 2, 1888. | 32 | S | ........ | Multitudinous subserous fibro-myomata of uterus with a development under left broad lig. size of woman's head. | Discovered 5 years ago. | Enucleation from broad lig. and extraperitoneal treatment of stump after Hegar Bladder dissected from anterior surface. | None except in broad lig. and to bladder. | Yes, tube above and iodoform guaze below stump. | St. Luke's Hospitai. | R | .................. | .................. | Chicago Gyn. Soc., May, 1888. |

### Vaginal Hysterectomy.

| No. | Name, and Date of Operation. | Age. | Married or Single. | No. of Children. | Pathological Condition or Symptoms necessitating Operation. | Duration of Disease. | Nature of Operation. | Adhesions. | Drainage. | Hospital or Private. | Recovery or Death. | Complications before or after. | Remarks and Subsequent History. | Elsewhere Reported. |
|---|---|---|---|---|---|---|---|---|---|---|---|---|---|---|
| 1 | Mrs. St—n. August 3, 1887. | 29 | M | 1 | Carcinoma of cervix. | Over a year. | Multiple ligatures of stumps with silk; catgut ligature about vaginal incision. Left vaginal and peritoneal wound open. | No | Iodoform gauze. | St. Luke's Hospital. | R | Cervix was amputated several mos. before. | No return. In good health. | Chicago Medical Soc., 1887. |
| 2 | Miss Ph—ps. Dec. 7, 1887. | 57 | S | ........ | Papilloma of cervix and posterior vaginal wall, undergoing sarcomatous degeneration. | Discovered 1 year before. | Three hæmostatic forceps to each broad ligament. Catgut ligature to vessels about vaginal incision. | No | Iodoform gauze. | Woman's Hospital. | R | .................... | Disease returned in two months; death in nine or ten mos. | Chicago Gynæcological Society, Dec., '88. |
| 3 | Mrs. G—n. Jan. 5, 1888. | 47 | M | 2, last 25 years ago. | Fibro-sarcoma of whole uterus, with ulceration of cavity. Uterus size of small fist. | 8 or 10 years. | Multiple silk lig .ture of stump. Catgut and two hæmostatic forceps below. Wound open. | No | Iodoform gauze. | Woman's Hospital. | R | Was curetted four times, viz.: 6½, 4½, 2 years and 1 month before operation. Thickening of right broad ligament. | So far, well. ....... | Chicago Gynæcological Society, Mar., '88. |
| | Mrs. Gold—t. March 4, 1888. | 55 | M | 2, oldest 17 years | Adenoma of fundus and posterior uterine wall undergoing cancerous degeneration. | Unknown, but over a year. | Retroverted uterus and applied multiple ligatures. Wound left open. | No | Iodoform gauze. | Woman's Hospital. | R | Cystocele before and after. | Perfectly well, except cystocele. | Chicago Gynæcological Society, Mar., '88. |
| 5 | Mrs. Florence J—s. Mar. 25, 1888. | 43 | M | 4, oldest 19 years | Three small interstitial fibro-myomata. Incurable stenosis. Several years of suffering and ineffectual local treatment. | Several years. | Multiple ligatures. Catgut to lower vessels. | No | Iodoform gauze. | St. Luke's Hospital. | R | .................... | Cured. ............ | Chicago Gynæcological Society, April, '88. |
| 6 | Mrs. O'B—n. May 17, 1888. | 25 | M | 2, younger 2 years old. | Cervical carcinoma involving posterior wall of uterus, to int. os, and entire thickness. | Not well since birth of last child. | Anteverted uterus and applied a medium and a large sized pair of forceps to each broad ligament. Catgut below. Took out left ovary with ligament cut short. | Loop of intestine tied off and separated. Left ovary embedded in lymph. | Iodoform gauze. | Woman's Hospital. | R | Left ovary enlarged and adherent to bottom of pelvis. Its ligature sloughed out in three months. | No sign of a return so far. | Chicago Gynæcological Society, May, '88. |
| | Miss McN. June 9, 1888. | 42 | S | ........ | Subserous fibro-myoma of posterior wall of cervix, size of goose egg. | A year or longer. | Multiple ligature of broad ligament. Catgut for lower vessels. | No | Iodoform gauze. | St. Luke's Hospital. | R | Unilocular cystoma of left ovary also removed, leaving ligature hanging in vagina. | Cured............. | Chicago Medical Society, June, 1888. |

### Exploratory Laparotomies.

| No. | Name, and Date of Operation. | Age. | Married or Single. | No. of Children. | Pathological Condition or Symptoms necessitating Operation. | Duration of Disease. | Nature of Operation. | Drainage. | Hospital or Private. | Recovery or Death. | Complications Before or After. | Subsequent History and Remarks. |
|---|---|---|---|---|---|---|---|---|---|---|---|---|
| 1 | Mrs. Dr. —. Oct. 7, 1888. | 30 | M | One, premature. | Chronic salpingitis, ovaritis and retroversion with fixation. Failure of prolonged course of treatment to relieve pain and restore her to usefulness. | About 6 years. | Small median incision and separating adhesion of uterus to rectum. Could not release the tubes nor find the ovaries. | No | Woman's Hospital. | Recovery. | Weak heart, almost died from ether. Had a pelvic abscess discharge into rectum at beginning of disease. | Is now somewhat better than before the operation. |
| 2 | Mrs. At—l. Dec. 21, 1887. | 28 | M | 0 | Menorrhagia, pains. Failure of mental and physical vigor, unrelieved by treatment. Apparent enlargement of tubes. | Several years. | Median incision about 3 inches long. Found the intestines matted about tubes, and the hæmorrhage so profuse upon the least attempt at separation that I tied the bleeding vessels and desisted. | Glass tube for 3 days. | Woman's Hospital. | Death at end of 8th day, of heart failure. | Weak heart. Came near collapsing several times on operating table. Pulse always above 100. | Died at 8 a. m. while having her shoulders raised to take nourishment. Had had an unusually smooth recovery and felt perfectly well just before being raised. |
| 3 | Mrs. W. G. McC—. Mar. 24, 1888. | 26 | M | 0 | Chronic incurable pelvic symptoms referable to appendages and incurable by prolonged treatment. Mind slightly affected. | Several years. | Median incision about 3 inches. Parovarian cyst of left broad lig. size of goose egg. R. appendages buried in lymph, and could not be separated. Separated a few adhesions | Glass tube for 3 days. | St. Luke's Hospital. | Recovery. | Piece of omentum size of Lima bean got into drainage tube and was torn out in removing it with considerable pain, but no after effects. | Husband thinks her mind greatly improved. |

### Summary.

| | Whole No. | Recovered. | Died. | Per cent. Recovery. | Per cent. Deaths. |
|---|---|---|---|---|---|
| Abdominal Ovariotomies | 6 | 4 | 2 | 66.66 | 33.33 |
| Vaginal Ovariotomies | 2 | 2 | 0 | 100.00 | ........... |
| Abdominal Oöphorectomies | 15 | 13 | 2 | 84.62 | 15.38 |
| Vaginal Oöphorectomies | 12 | 12 | 0 | 100.00 | ........... |
| Abdominal Hysterectomies | 3 | 1 | 2 | 33.33 | 66.66 |
| Vaginal Hysterectomies | 7 | 7 | 0 | 100.00 | ........... |
| Exploratory Incisions | 3 | 2 | 1 | 66.66 | 33.33 |
| Total | 48 | 41 | 7 | 82.92 | 17.08 |

